# Safety and effectiveness of an herbal decoction (modified Saengmaeksan) in hypertensive patients: Protocol for a real-world prospective observational study

**DOI:** 10.1371/journal.pone.0316276

**Published:** 2025-01-17

**Authors:** Nahyun Cho, Hobin Moon, Kyung-Min Shin, Byoung-Kab Kang, Jungtae Leem, Changsop Yang

**Affiliations:** 1 Department of Diagnostics, College of Korean Medicine, Wonkwang University, Iksan, Republic of Korea; 2 Kyunghee Bichedam Clinic of Korean Medicine, Seoul, Republic of Korea; 3 KM Science Research Division, Korea Institute of Oriental Medicine, Daejeon, Republic of Korea; 4 Research Center of Traditional Korean Medicine, College of Korean Medicine, Wonkwang University, Iksan, Republic of Korea; SDM College of Medical Sciences and Hospital, Shree Dharmasthala Manjunatheshwara SDM University, INDIA

## Abstract

**Objective:**

Hypertension, a common chronic disease, often leads to serious complications. While conventional management relies on antihypertensive drugs, which can cause side effects and adherence issues, alternative treatments like herbal medicine are gaining attention. This study examines the efficacy and safety of modified Saengmaeksan, an East Asian herbal remedy, in treating hypertension.

**Methods:**

This single-arm, prospective, observational study will be conducted at Kyunghee Bichedam Korean Medicine Clinic from October 23, 2023 to August 30, 2024, enrolling 30 hypertensive patients. Over 12 weeks, participants will undergo 4 visits, receiving modified Saengmaeksan twice daily for 8 weeks, with a subsequent 4-week follow-up. Primary outcome is the change in systolic blood pressure from the baseline to week 8. Secondary outcomes include diastolic blood pressure changes, radial artery tonometry, and quality of life evaluations. Safety assessments will include monitoring hematologic parameters and adverse events. Data will be analyzed using an ANCOVA model for adjusting confounders.

**Discussion:**

Modified Saengmaeksan has shown potential for lowering blood pressure in clinical settings, supported by animal and cell studies. However, human studies are scarce. This research will employ radial artery tonometry to analyze blood pressure comprehensively, exploring Saengmaeksan’s hemodynamic effects. The study’s goal is to support the approval of modified Saengmaeksan as a hypertension treatment by the South Korean Food and Drug Administration and to promote the industrialization of traditional herbal medicine in managing hypertension. The findings will provide essential data for future clinical research, aiding in feasibility assessments and sample size determinations for randomized controlled trials.

## 1 Introduction

Hypertension is one of the most common chronic diseases in primary healthcare and one of the leading causes of preventable death. Hypertension is a major risk factor for cardiovascular, cerebrovascular, and renal diseases, which, if left untreated, can lead to serious complications and target organ damage [1–3]. From a long-term perspective, hypertension has become a significant challenge in public health, as it is a major contributor to disability-adjusted life years and high mortality rates. Therefore, appropriate management of hypertension is increasingly recognized as a crucial aspect of public health [4,5]. In 2019, it was estimated that approximately 1.2 billion people worldwide were living with hypertension, [6] and by 2025, it is estimated that 29.2% of the world’s population will have hypertension [7]. In Korea, the prevalence of hypertension in adults aged 30 years and older was 33% in 2018, and it has been increasing slightly in recent years [8]. According to a study in 2020, the economic burden of hypertension globally amounted to hundreds of billions of dollars [9]. The management of hypertension is becoming increasingly important to address the growing socioeconomic burden of hypertension and to improve the quality of life of individuals.

The administration of oral antihypertensive agents, such as diuretics, beta-blockers, calcium channel blockers, and ACE inhibitors, is the standard approach for managing hypertension [10]. Nevertheless, issues may arise, such as inadequate blood pressure control leading to the need for dose escalation, discontinuation of medication due to drug-related side effects or poor medication adherence [11]. Even when blood pressure is controlled within the normal range, patients may still exhibit a high incidence of cardiovascular diseases and mortality [12]. Furthermore, the unavoidable side effects of antihypertensive drugs (e.g., headaches, dizziness, orthostatic hypotension, and sexual dysfunction [13]), as well as symptoms accompanying hypertension and resulting decreases in quality of life, remain challenges faced by all. Therefore, there is a need for alternative treatment approaches to address various unmet healthcare demands, such as low adherence to hypertension treatment or the occurrence of side effects in the management of hypertension [14].

A variety of alternative treatments for prehypertensive and hypertensive patients, such as exercise, meditation, biofeedback approaches, qigong, etc. [15], and especially in East Asia, acupuncture [16,17], moxibustion [18], cupping [19], and herbal treatments [20], have long been utilized in clinical practice. In ancient China, hypertension was viewed as a category of "dizziness, headache, fatigue, rapid pulse, etc." according to its typical symptoms and signs [21]. To treat these symptoms, decoctions were made using a combination of herbal medicines. Among these, traditional herbal remedies such as Orengedoku-to (Huang Lian Jie Du Tang) [22], Samhwangsasim-tang (San Huang Xie Xin Tang) [23], Jingansikpungtang (Zhen Gan Xi Feng Tang) [24], Banhabaekchulcheonma-tang (Ban Xia Bai Zhu Tian Ma Tang) [25], and herbal medicine for pacifying the liver to subdue yang [26], which are often used to "soothe the liver and extinguish wind" or "clear heat," have been frequently researched for their potential in lowering blood pressure. There are also reports of herbal medicines improving the quality of life and reducing side effects in patients with hypertension [27]. However, although Saengmaeksan (SMS) and modified Saengmaeksan (mSMS), which utilize a strengthened blood vessel strategy in East Asian traditional medicine (EATM) theory, are also commonly used to treat hypertension in clinical practice, there are fewer clinical studies on mSMS [28]. Based on clinician’s experience, EATM classics, and experimental results [29], the composition of medicinal herbs in the herbal prescription mSMS has changed as follows: 5.33 g of Puerariae Radix, 5.33 g of Platycodonis Radix, 5.33 g of Liriopis seu Ophiopogonis Tuber, 2.67 g of Dioscoreae Rhizoma, 2.67 g of Coicis Semen, and 2.67 g of Schisandrae Fructus. With the proven vasodilatory effects of Puerariae Radix, it is predicted that it would have a positive impact on lowering blood pressure [30,31]. Animal studies have shown significant blood pressure lowering effects with the administration of mSMS [29]. However, despite its widespread clinical use, there is a lack of evidence in clinical studies.

Therefore, this study aims to conduct a real-world, prospective observational single arm study to assess the clinical effectiveness and safety of mSMS, a key prescription for the treatment of hypertension that is widely utilized in clinical practice. In addition to measuring blood pressure and relevant indicators, we intend to also assess hemodynamic mechanisms by measuring pulse waves. Furthermore, we aim to investigate changes in the quality of life of hypertensive patients after treatment. Through this research, we will accumulate fundamental information necessary for determining effect sizes, appropriate treatment durations, and outcome variables for future randomized controlled studies on mSMS. This study will serve as the foundation for subsequent research.

## 2 Objectives

### 2.1 Primary objectives

The primary objective of this study is to evaluate the change in mean sitting systolic blood pressure (MSSBP) following oral administration of mSMS in adult hypertensive patients in a real-world setting.

### 2.2 Secondary objectives

#### 2.2.1 Blood pressure

- To determine the change from baseline in MSSBP and mean sitting diastolic blood pressure (MSDBP) at each assessment time point.
- To determine the proportion of patients with MSSBP/MSDBP <140/90 mmHg at the end of treatment.
- To observe the response rate of blood pressure at the end of treatment compared to the baseline (the proportion of patients who experience a mean systolic blood pressure reduction of 20 mmHg or more or a diastolic blood pressure reduction of 10 mmHg or more).

#### 2.2.2 Radial artery tonometry device parameters

At each endpoint, we will further observe the changes in individual pulse strength, pulse depth, pulse rate, pulse shape, augmentation index (R-AI), APG type, SBP, DBP, Pulse pressure (PP), Heart Rate (PR), Stroke Volume, Cardiac output, systemic vascular resistance index (SVRI) through the radial artery tonometry device parameters from baseline.

#### 2.2.3 Quality of life

We will determine the change in pre- and post treatment quality of life in hypertensive patients receiving mSMS.

#### 2.2.4 Covariates

We will identify covariates, including sex, age, BMI, duration of hypertension, comorbidities, baseline blood pressure, antihypertensive medication, and the type of antihypertensive medication, that may significantly impact treatment efficacy.

### 2.3 Safety

Adverse events related to mSMS use will be recorded by the Kyunghee Bichedam Korean Medicine Clinic and a safety evaluation will be conducted. Adverse effects (AEs) related to the intake of mSMS, such as indigestion, heartburn, and palpitations, will be collected through voluntary reports from study participants at each visit. Additionally, safety evaluation will be conducted at each visit by assessing vital sign measurements and hematological tests.

## 3 Methods

### 3.1 Study design/setting

This is a prospective, single-arm, single-center, prospective observational study with no control group; therefore, no blinding or randomization is needed. The study is being conducted at Kyunghee Bichedam Korean Medical Clinic (28 Nonhyeon-ro 10-gil, Gangnam-gu, Seoul, South Korea). Recruitment is scheduled to begin in October 23, 2023 and end in August 31, 2024.

The protocol ver1.1 of this study was approved by the Public Institutional Review Board Designated by the Ministry of Health and Welfare 2nd committee (P01-202309-01-027) and registered in the Korean Clinical Trials Registry (trial registration number: KCT0008920). The reporting of this study complies with the Standard protocol items recommended for intervention trials (SPIRIT) checklist (S1 File).

### 3.2 Ethics issues

While participating in the study, participants should expect to experience minimal discomfort, comparable to that encountered with routine medical interventions and tests. To address this, the researcher will communicate professionally with the participant. The researcher will also provide detailed answers to any questions the participant may have about the study during the study. During the recruitment of study participants, the landline number of Kyunghee Bichedam Korean Medicine Clinic and the phone number of the researcher in charge were provided to the study participants. No compensation will be provided to participants for this study other than a small transportation allowance, a decision that was approved by the IRB to minimize financial coercion and maintain voluntary participation. This study is an observational study following routine clinical protocols, and there are no additional risks associated with participation in the study itself. However, for AEs that may occur within the scope of routine clinical care, compensation will be provided according to the malpractice liability insurance regulations of the Kyunghee Bichedam Korean Medicine Clinic. The protocol of this study declares that the privacy of the research subjects will be protected and will be conducted with respect to the individual subjects in accordance with the Declaration of Helsinki. The source data will be stored securely at Kyunghee Bichedam Korean Medicine Clinic. This study protocol was approved by the Public Institutional Review Board (IRB) designated by the Ministry of Health and Welfare on September 13, 2023 (no. P01-202309-01-027), and the risk of data leakage will be prevented by a well-established data storage and management plan. The source data will be securely stored at Kyunghee Bichedam Korean Medicine Clinic.

### 3.3 Eligibility criteria and study enrollment

#### 3.3.1 Inclusion criteria

1. Adult men and women aged 19 or older but less than 75 years of age.
2. Those whose blood pressure measured in the reference arm (selected as the reference arm at the time of screening) meets the following criteria:
  A. In the absence of antihypertensive medication use: 140 mmHg ≤ MSSBP < 180 mmHg; or
  B. In the presence of antihypertensive medication use: 130 mmHg ≤ MSSBP < 180 mmHg [32].
3. Individuals who receive detailed explanations about this observational study, fully understand it, and voluntarily consent to the provision of medical information, as well as the collection and utilization of personal information.

#### 3.3.2 Exclusion criteria

1. Individuals who, with at least a 2-minute interval between measurements on both arms, exhibit a difference of 20 mmHg or more in sitting systolic blood pressure (SSBP) and 10 mmHg or more in sitting diastolic blood pressure (SDBP) over three consecutive measurements.
2. Individuals with MSSBP ≥ 180 mm Hg or MSDBP ≥ 110 mm Hg in the arm selected as the reference arm at screening.
3. Individuals with known liver or kidney disease that requires treatment.
4. Patients with secondary hypertension.
5. Individuals who have been diagnosed with a serious cardiovascular condition: myocardial infarction, angina, severe arrhythmia, etc.
6. Individuals who have been diagnosed or treated for a psychoneurological disorder, including depression, within 2 months of their visit.
7. Any other person whose Korean Medicine doctor with over 10 years of clinical experiencedetermines that mSMS is not appropriate for them.

#### 3.3.3 Recruitment

The study will be conducted among patients visiting the Kyunghee Bichedam Korean Medicine Clinic who express their intention to participate after reviewing the recruitment notice posted within the facility. Upon obtaining patient consent, a screening evaluation will be performed to assess eligibility based on inclusion and exclusion criteria before enrolling them in the study. When obtaining consent, patients will be informed about the research purpose and methods, and they will be given assurance that there is no additional financial burden for the patient regarding treatment and examinations. Only those who voluntarily provide written consent will be registered for the study.

### 3.4 Usual standard treatment protocol

Enrolled study participants will receive an mSMS according to the usual standard treatment protocol used in the actual clinical hypertension treatment process at Kyunghee Bichedam Korean Medical Clinic for a duration of 8 weeks (56 days). Research subjects will be administered mSMS, a prescription in the form of a 70% alcohol extract, twice daily, either before meals or between meals. Each mSMS packet (60 ml) is in the form of an alcohol-extracted decoction obtained from the following herbal ingredients: 5.33 g of Puerariae Radix, 5.33 g of Platycodonis Radix, 5.33 g of Liriopis seu Ophiopogonis Tuber, 2.67 g of Dioscoreae Rhizoma, 2.67 g of Coicis Semen, and 2.67 g of Schisandrae Fructus, totaling 24 g (per day). As this study is observational, no restrictions will be imposed on concurrent intervention and concomitant medications during the administration of mSMS. However, all concurrent treatments will be carefully documented to control for potential confounding variables during the analysis phase. Additionally, since mSMS has a blood pressure-lowering mechanism different from that of conventional antihypertensive drugs, we do not plan to adjust the dosage of mSMS even if blood pressure sufficiently decreases during the study period. Considering past clinical experience, the likelihood of hypotension occurring is minimal, and if it does occur, it will be managed in the same manner as the occurrence of other side effects (Table 1).

**Table 1:** Ingredients of modified Saengmaeksan.

Ingredients (Pinyin)	Scientific Name (Plant sources)	English Name	Used part of Plant
Puerariae Radix (葛根, Gegen)	*Pueraria montana* var. *lobata* (Willd.) Maesen & S.M.Almeida ex Sanjappa ex Sanjappa & Predeep	Pueraria Root	Root
Platycodonis Radix (桔梗, Jiegeng)	*Platycodon grandiflorus* (Jacq.) A.DC.	Platycodon Root	Root
Liriopis seu Ophiopogonis Tuber (麥門冬, Maidong)	*Liriope platyphylla* Wang et Tang	Liriope Tuber	Tuber
Dioscoreae Rhizoma (山藥, Shanyao)	*Dioscorea polystachya* Turcz.	Dioscorea Rhizome	Rhizome
Coicis Semen (薏苡仁, Yiyiren)	*Coix lacryma-jobi* var. *ma-yuen* (Rom.Caill.) Stapf	Coix Seed	Seed
Schisandrae Fructus (五味子, Wuweizi)	*Schisandra chinensis* (Turcz.) Baillon	Schisandra Fruit	Fruit

### 3.5 Data collection

**Fig 1:**
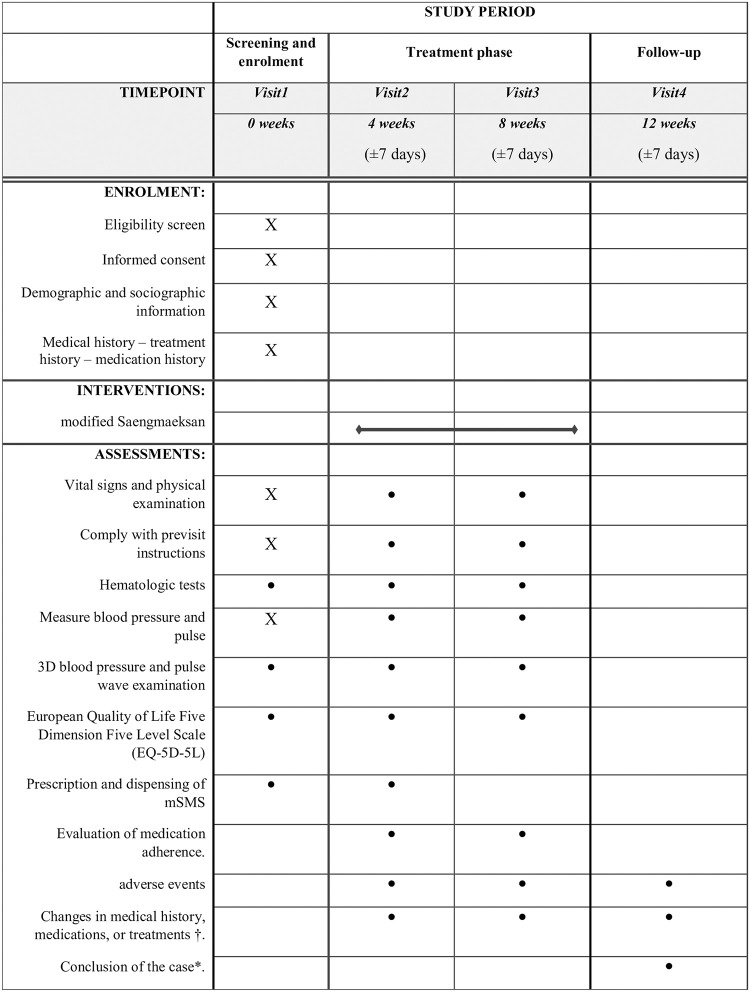
Schedule of enrolment, interventions, and assessments. * Case conclusion: Collect case conclusion if the visit is terminated or there is a dropout. † Change in medical history, medication history, and treatment history: Collect items if new medical history, medication, or treatment occurred after enrollment (baseline). ● Only subjects who pass the eligibility criteria will be tested.

The data collection and follow-up schedule is presented in Fig 1.

#### 3.5.1 Screening and enrollment phase

Subjects will be screened for inclusion/exclusion criteria. Information and tests collected during the screening phase will include demographics, sociodemographics, vital signs, physical examination, compliance with previsit instructions, medical history, treatment history, medication history, blood pressure and pulse measurements. Participants on antihypertensive medications will continue their regimen, while those not previously on such medication will remain off it unless otherwise indicated. Any changes in medication, including antihypertensive drugs, will be recorded in the eCRF at each visit. Patients who meet the inclusion/exclusion criteria will be enrolled in the study and will undergo hematologic testing, 3D blood pressure and pulse wave examination, and European Quality of Life Five Dimension Five Level Scale (EQ-5D-5L) questionnaire testing. Information regarding the prescription and dispensing of mSMS will be collected.

#### 3.5.2 Treatment phase

After screening, patients will visit at 4 weeks (±7 days) and 8 weeks (±7 days) after enrollment (from baseline) to have their vital signs checked, undergo a physical examination and confirm compliance with previsit instructions. Hematologic examination, blood pressure and pulse measurement, 3D blood pressure and pulse wave examination, EQ-5D-5L questionnaire examination, confirmation of the dosage of mSMS, adverse events, medical history, medication history, and treatment history that have occurred since enrollment in the study will also be assessed. In addition, we will collect information on the prescription and dispensing of mSMS at visit 1 and visit 2, when mSMS is newly prescribed, and check medication at visits 2 and 3.

#### 3.5.3 Follow-up visit

We will follow-up by phone 12 weeks (±7 days) after study enrollment (postbaseline) to identify adverse events and changes in medical history, medication history, and treatment history.

### 3.6 Outcome and covariates measurement

#### 3.6.1 Demographic and sociodemographic information and general information

Information will be collected at participant screening, including date of birth, sex, physical measurements such as height and weight, vital signs such as pulse and temperature, past medical history, surgical history, medications, and compliance with pre-visit instructions, which will be recorded as Yes or No. Pre-visit instructions include fasting for 8 hours before the visit, no smoking the day before the visit, no alcohol consumption the day before the visit, no caffeine intake the day before the visit, and scheduling the visit between 8 AM and 12 PM.

#### 3.6.2 Hematologic tests

The Samsung LABGEO PT10 (Samsung Electronics, Suwon, Korea) analyzer will be utilized to measure the following parameters: aspartate transaminase (AST), alanine transaminase (ALT), gamma glutamyl peptidase (GGT), total bilirubin, blood urea nitrogen (BUN), creatinine, total cholesterol, and glucose.

#### 3.6.3 Blood pressure and pulse measurements

Measurement of MSSBP, MSDBP, and pulse will be conducted using the electronic blood pressure monitor Omron HEM-9000AI and SphygmoCor (AtCor Medical, Sydney, Australia). During the screening, MSSBP and MSDBP will be assessed by taking three blood pressure readings on both arms while the participant will be in a seated position.. Subsequently, the arm with the higher average systolic blood pressure will be selected as the reference arm. Once the reference arm is determined, blood pressure measurements will be taken from the reference arm identified during the screening visit [32].

#### 3.6.4 3D blood pressure pulse wave examination

DMP-Life plus (Daeyomedi, Ansan, Korea), a radial artery tonometry device, will be utilized. The DMP-Life plus is a new blood pressure pulse analyzer (BPPA) which utilizes tonometry with piezoresistive array sensor array technology, and unlike conventional systems that only measure BP, it is able to analyze waveforms in three dimensions with array sensors [33]. The following items will be collected:

- Pulse Strength Measurement and Level (Unit: gf)
- Pulse Depth measurements and Level (unit: gf/cm^2)
- Pulse rate measurements and level (in beats)
- Pulse shape measurements and level (unit: step)
- Because the radial artery R-AI is affected by heart rate, we use a value that is normalized to a heart rate of 75 bpm ((R-AI) @75) (unit: %) [34]
- APG type
- SBP (unit: mmHg)
- DBP (unit: mmHg)
- PP (unit: mmHg)
- PR (unit: beats/min)
- Stroke Volume (once) (unit: mL/beat)
- Cardiac output (1 min) (unit: L/min)
- SVRI (unit: dyn*s/cm^5/m^2)

#### 3.6.5 European Quality of Life Five Dimension Five Level Scale (EQ-5D-5L)

The EQ-5D-5L is a patient-reported outcome measure of current health status and quality of life and consists of five items (mobility, self-care, activities of daily living, pain/discomfort, and anxiety/depression). Each question is rated on a 5-point equator (1: no problem, 2: mild problem, 3: moderate problem, 4: severe problem, 5: extreme problem) [35]. The Korean version of the EQ-5D-5L will be used in this study [36].

### 3.7 Data management

All documents related to study participants will be recorded and distinguished by the participant identification code, not the participant’s name. Relevant data will be stored on a computer with restricted access through encryption, accessible only to authorized personnel. The distribution of questionnaires and examinations for data acquisition will be conducted exclusively by researchers at Kyunghee Bichedam Korean Medical Clinic. The researchers have received training for the investigation and examination processes. All data collection will be carried out through the establishment of an online eCRF. The collected data will be stored through an electronic data capture (EDC) system called mytrial, managed by the National Institute for Korean Medicine Development (NIKOM). Collected data will be available for use after obtaining permission for public purposes. Trained and qualified researchers entered the primary data into the eCRF to build the database. Information in the online database can be modified and updated in a timely manner. Additionally, data can be exported in real time for quality control and statistical analysis purposes.

Monitoring is conducted by designated Clinical Research Associate from the Korea Institute of Oriental Medicine, independent of investigators or sponsors. Associates will be responsible for ensuring compliance with the clinical research protocol, appropriate and accurate data collection, verification of informed consent from study participants, collection and reporting of (serious) adverse events, and oversight of investigational drug management for the clinical study. In this study, monitoring consists of initiation visits, routine monitoring visits, and close-out visits. To verify the accuracy of the initial data collection and ensure compliance with the protocol, the first monitoring visit will be conducted within 3 weeks of the initial study participant registration. Subsequent routine monitoring visits will be conducted when the number of screened study participants exceeds 50% of the target, again when it reaches 100% of the target, and periodically after the last participant is enrolled to check for dropouts and to assess the status of visits 2, 3, and follow-up.

### 3.8 Adverse events

AEs refer to undesirable and unintended signs, symptoms, or diseases that occur in study participants during clinical research. While the risk of expected AEs related to the consumption of mSMS in this study is low, common AEs, similar in level to those in routine clinical practice, such as indigestion, heartburn, and palpitations, may occur.

AEs will be collected through the judgment of researchers or voluntary reports from study participants at each visit (Visit 2 and Visit 3). During the follow-up period, the occurrence of AEs was verified via telephone. This study is covered by clinical research insurance, and a 24-hour contact number was provided through the patient recruitment notice. It was clearly stated in the IRB-approved study protocol that in the event of any issues, the medication would be discontinued at the discretion of the principal investigator, and necessary medical actions would be taken. Serious adverse events (SAEs) will be promptly reported through the e-IRB system, following the provisions of Chapter 25, Article 4, in the Standard Operating Procedures of the Public Institutional Review Board. The definition of severe adverse events aligns with the Standard Operating Procedures of the Public Institutional Review Board.

Ultimately, the reporting includes the incidence rate of adverse events, the incidence rate of adverse events that resulted in dropouts, and the incidence rate of severe adverse events. This incidence rate is presented in two categories: the incidence rate for all adverse events and the incidence rate for adverse events related to mSMS intake. Additionally, an assessment of vital sign measurements and hematological tests will determine normal/abnormal results, and statistical tests will be performed only on those deemed clinically significant by the clinician.

### 3.9 Withdrawal and dropout criteria

Although participants will have voluntarily agreed to participate in the study, they may withdraw their consent at any time during the study and will not be penalized for withdrawing their consent. If a participant withdraws or drops out of the study, the data collector will record the main reasons for the dropout, and the participant’s personal information and related data will not be used for research analysis and will be destroyed immediately.

Subjects may be withdrawn from the study for the following reasons:

- the subject or the subject’s legal representative requests discontinuation of the study due to unsatisfactory treatment effects during the observational study period, and the subject withdraws consent to participate in the study;
- violations of inclusion criteria or meeting exclusion criteria;
- even if a participant agrees to take part in the study and meets the eligibility criteria, they will be excluded if they have never take mSMS during the observational study period;
- if a subject has an SAE caused by herbal medicine or if the AE makes it difficult to continue the study;
- if the prescribed herbal medicine changes during the study;
- violation of the research protocol by a participant;
- failure to follow up with the subjects during the study period; and
- if the researcher determines that it is not appropriate for the participant to continue to participate in the study.

### 3.10 Sample size calculation

According to existing studies, preliminary studies usually require more than 12 participants [37]. In this study, our observational study will be conducted with a sample size of approximately 20 participants. We calculated the expected number of participants by considering that there are approximately 10 patients per month who take mSMS for hypertension treatment at Kyunghee Bichedam Korean Medical Clinic. Considering a dropout rate of 33%, 30 people will be recruited to obtain a sample size of 20.

### 3.11 Statistical analysis

All analyses will be performed using SAS Version 9.4 (SAS Inc., Cary, NC), and all statistical tests will be two-tailed at a significance level of 5%, unless otherwise defined. Demographic and baseline information will be described as n (%) for categorical data and mean±SD for continuous data.

Data for the effectiveness evaluation will be analyzed using the Full Analysis Set as the primary analysis. It is composed of groups that follow the intention-to-treat principle and have at least one measurement of the primary efficacy endpoint after the administration of mSMS. In case of missing values for FAS in the efficacy evaluation, statistical analysis will be performed by applying the last observation carried forward method; otherwise, statistical analysis will be performed according to the original data.

Effectiveness evaluation of continuous variables is subject to paired t test or Wilcoxon signed rank test depending on whether normality is satisfied, and categorical variables will be subject to McNemar test or McNemar Exact test.

For MSSBP, MSDBP, 3D blood pressure pulse wave, and EQ-5D-5L scores, patients showing improvement at visits 2 and 3 compared to baseline will be considered to have a treatment effect. The proportion of respondents who demonstrated improvement is then presented for each outcome variable. However, since this is a feasibility study without a control group, there will be limitations in determining the effectiveness. To control for potential confounding variables that could influence blood pressure, we aimed to collect data on the following covariates and control for them during the statistical analysis process.

- Information about whether the participants took medication for hypertension was collected.
- If participants are taking medication to treat hypertension, they will be instructed to notify the researcher of any changes in their medication or dosage.
- Participants will be asked to try to keep their lifestyle the same, including exercise and diet.

This data will be analyzed using an ANCOVA model with the following covariates: sex, age, BMI, duration of hypertension, comorbidities, baseline mean systolic blood pressure, and whether and what type of antihypertensive medication is being taken.

Primary outcome

- The change in MSSBP from baseline (visit 1) to the end of treatment (visit 3) using the Omron Healthcare HEM-9000AI blood pressure monitor.

#### 3.11.1 Secondary outcomes

*3*.*11*.*1*.*1 Blood pressure*

- Change in MSSBP from baseline (visit 1) to visit 2;
- change in MSDBP from baseline (visit 1) to visit 2 and visit 3;
- proportion of patients with MSSBP/MSDBP <140/90 mmHg at the end of treatment [32]; and
- the blood pressure response rate at the end of treatment (visit 3) compared to the baseline (visit 1) (the proportion of patients who experience a mean systolic blood pressure reduction of 20 mmHg or more or a diastolic blood pressure reduction of 10 mmHg or more) [32].

*3*.*11*.*1*.*2 Radial artery tonometry device parameters*

- Change from baseline (visit 1) to visit 2 and visit 3 in individual parameters for 3D blood pressure pulse wave examination.

*3*.*11*.*1*.*3 Quality of life*

- Change in European Quality of Life Five Dimension Five Level Scale (EQ-5D-5L) questionnaire score from baseline (visit 1) to visit 2 and visit 3.

#### 3.11.2 Analysis of safety evaluations

Statistical analysis of safety will be conducted using the safety set. The safety set is composed of subjects who received at least one dose of mSMS and had at least one safety-related follow-up. AEs, SAEs, and serious adverse drug reactions will be presented as n (%), and differences between treatment points will be tested by the McNemar test or McNemar Exact test.

## 4 Discussion

Existing observational studies of herbal medicines are limited by the fact that they are composed of multiple traditional medicine interventions and cannot evaluate the effect of herbal medicines alone. Therefore, the present study was designed with the aim of conducting a prospective observational study in hypertensive patients to determine the effect of herbal medicine alone.

### 4.1 Predicted therapeutic mechanisms of mSMS

SMS, a traditional herbal medicine, is composed of Ginseng Radix (Renshen), Liriopis seu Ophiopogonis Tuber (Maidong), and Schisandrae Fructus (Wuweizi). Clinically, SMS has been used to treat heart failure [38] and angina pectoris [39], and a systematic review revealed that it can significantly improve symptoms of ischemic stroke [40]. In rats with induced cardiomyopathy, a study showed cardiac function improvements following injection of SMS [41], and cellular studies have shown that it inhibits cerebral ischemia‒reperfusion injury [42].

For hypertension, blood pressure-lowering effects in rats after administration of SMS [43] and reduced NO production in aortic smooth muscle cells in cellular experiments [44] However, there are no direct human studies of blood pressure-lowering effects of SMS in clinical practice. There are no clinical study results available for ’mSMS’, which is based on SMS and involves the addition or subtraction of herbal ingredients. Particularly, in the case of Pueraria Root, it is extensively utilized in various antihypertensive herbal formulations due to its vasodilatory properties, and it is expected to exhibit a synergistic antihypertensive effect when combined with SMS [30,31,45]. Cellular and animal experiments have confirmed its vasodilation and blood pressure-lowering effects [46,47]. Through systems pharmacology, it has also been reported that Pueraria root can influence various modules, such as proliferation, apoptosis, and inflammation, which could impact blood pressure reduction [48]. The mSMS used in this study has demonstrated antihypertensive effects in many hypertensive patients in clinical settings; however, there is no prospective research evidence. Through this study, we aim to establish clinical evidence and subsequently explore the experimental basis and mechanisms of mSMS using a bedside-to-bench approach, i.e., reverse pharmacology. By including patients who meet hypertension criteria without taking antihypertensive drugs and those whose BP remains uncontrolled despite taking these medications, our study aims to explore the efficacy and safety of mSMS in supplementing existing antihypertensive treatments. This approach addresses the practical challenges observed in real-world clinical settings and seeks to provide insights into how mSMS can potentially enhance blood pressure management in patients with poor medication adherence or inadequate response to current treatments. Ultimately, this study will provide clinical evidence for the utilization of mSMS as a new treatment option in the management of hypertension.

### 4.2 Design features of the study and implications for future research

Recently, radial artery tonometry using piezoresistive array sensors has been offered as a noninvasive method of blood pressure monitoring and pulse waveform analysis [49,50]. In both Eastern and Western medicine, measuring the pulse at the radial artery and analyzing both the temporal and spatial characteristics of the pulse wave has long been a major diagnostic modality. Analyzing the morphology of the pulse wave can provide a great deal of information about cardiovascular risk factors, as it reflects hemodynamic features such as the elasticity and viscosity of the arterial wall, vascular resistance, compliance, and vascular stiffness [51]. Therefore, there is a growing need for systems that can accurately measure the detailed morphological characteristics of pulse waves [52]. In addition, noninvasive or minimally invasive techniques have become increasingly important for monitoring hemodynamic parameters in the clinical setting, and radial artery blood pressure pulse wave analysis methods that can be measured by simply attaching a diagnostic device have received increasing attention [53,54].

Another important advantage of pulse waveform measurement is that the sensor can be used to simultaneously and relatively accurately measure cardiac output (CO), systemic vascular resistance index (SVRI), and blood pressure [55]. Blood pressure is a physiologic measure that reflects cardiac output and circulatory resistance [56]. Therefore, classification based on CO and SVRI may play an important role in characterizing subtypes of hypertension and selecting treatment strategies [57]. In this regard, clinical indicators of hypertension require a detailed analysis of pulse waveforms as well as blood pressure.

In this study, in addition to standard blood pressure measurements, we will collect indicators related to pulse waveforms using 3D blood pressure wave analysis. Daeyo Meditech Co., Ltd. developed the DMP-Lifeplus model, a three-dimensional pulse imaging diagnostic device, which complies with the international standard ISO 18615 ‘General requirements for basic safety and essential performance of electric radial pulse tonometric devices’ [58,59]. The 3D radial artery tonometry device not only diagnoses simple pulse waveforms but also reflects the traditional active method of pulse diagnosis, which requires the examiner’s involvement in locating the radial artery and precise pressure adjustment [59]. Furthermore, the DMP-Lifeplus has been recognized for its utility through years of accumulated clinical research data [55,60,61]. It is the first traditional Korean diagnostic device to receive medical technology evaluation from the National Health Insurance Service (NHIS) and become a reimbursable medical procedure [62].

Hypertension not only affects physical condition but also affects mental health, such as anxiety and depression [63]. This decline in mental and physical health has been shown to lead to a decrease in quality of life [64]. The EQ-5D-5L is a well-known health-related quality of life (HRQoL) assessment tool commonly used worldwide [65–67], and it has been reported to improve discrimination between different levels of health [68]. Therefore, the EQ-5D-5L will be utilized in this study to accurately assess the quality of life of hypertensive patients.

By analyzing the outcomes collected by the test in subgroups, we aim to analyze the confounders that affect the blood pressure lowering effect of mSMS. We predict that the treatment effect will be different depending on blood pressure medication, comorbidities, baseline hypertension, and BMI, and we will perform additional subgroup analyses of these covariates. This will allow us to identify a more responsive responder group and adjust the inclusion and exclusion criteria for subsequent trials.

### 4.3 Significance of the study in the healthcare insurance system and healthcare-related regulation in Korea

This study is an outgrowth of the research on mSMS, which originated from research on public resource-based research projects. Public resource-based research projects support the process of securing intellectual property rights through animal studies, clinical research evidence, patenting, and eventually approval and coverage of a product through clinical trials [69]. In Korea, herbal medicines are used for various diseases, but the industry related to herbal medicines is underdeveloped, so it is slow to establish clinical trial evidence and CMC data for the industrialization of herbal medicines led by pharmaceutical companies [70]. Due to the small scale of the herbal medicine industry and legal deficiencies, there is limited activity in conducting phase 3 clinical trials for herbal medicine. Therefore, there are no cases where herbal medicine has undergone clinical trials, obtained approval from the Korean Food and Drug Administration, and is covered by national health Insurance for Korean Medicine practice. In addition, due to the abovementioned problems, pharmaceutical companies may decide that after successful clinical trials, prescriptions derived from herbal medicines will be covered only by conventional medicine doctors. These institutional and legal issues create a vicious cycle of high dissatisfaction among practitioners and low willingness to industrialize herbal prescription. To address the issues mentioned above, mSMS intends to take the following steps through government-funded research. Starting with a retrospective chart review, we conducted a prospective preliminary observational study, animal study, and CMC data construction.

If this study is successfully completed and subsequent phases II and III are completed, it will be the first study in Korea where a prescription developed through clinical experience will be covered by health insurance through the process of new drug development.

### 4.4 Strengths and limitations of the study

However, a limitation of this study is that it is a single-arm observational study with no control group, so data on efficacy may be limited. Another limitation is that the patients will be recruited from a single institution, and the sample size is only 30 patients, so there is a possibility of bias in the efficacy results. However, this study is an observational study to preliminarily explore the effectiveness of mSMS in treating hypertension, and observational studies can provide fundamental data for future randomized controlled trials [71]. Therefore, even though causality cannot be stated based on this observational study alone, this can be a study to explore the adherence, clinical course, safety, and feasibility of conducting a randomized controlled trial in the future.

### 4.5 Dissemination plan

The results will be registered on the public website of NIKOM, published in a peer-reviewed journal, and distributed electronically and in print, ensuring transparency and contributing to the scientific community regardless of the outcome. The results will also be used for conference presentations, made available on the National Clearinghouse for Korean Medicine (NCKM) website, and secondarily utilized in the National Institute for Korean Medicine Development database. Any formal presentation or publication of data collected from this study will be considered as a joint publication by the participating physician(s) and will follow the recommendations of the International Committee of Medical Journal Editors (ICMJE) for authorship.

## Supporting information

S1 File SPIRIT 2013 checklist. (DOC)

S2 File Study protocol for IRB English. (DOCX)

## Abbreviations

AE: adverse event
APG: accelerated photoplethysmograph
CO: cardiac output
CRF: case report form
EQ-5D-5L: European Quality of Life Five Dimension Five Level Scale
MSDBP: mean sitting diastolic blood pressure
mSMS: modified Saengmaeksan
MSSBP: mean sitting systolic blood pressure
PP: pulse pressure
PR: pulse rate
R-AI: augmentation index
SDBP: sitting diastolic blood pressure
SMS: Saengmaeksan
SSBP: sitting systolic blood pressure
SV: stroke volume
SVRI: systemic vascular resistance index
