# Supplementary material for: Safety and effectiveness of an herbal decoction (modified Saengmaeksan) in hypertensive patients: Protocol for a real-world prospective observational study
Source: PLoS One. 2025 Jan 17;20(1):e0316276. doi: 10.1371/journal.pone.0316276 (PMC11741599; doi:10.1371/journal.pone.0316276)
Supplement: S2 File — (DOCX) [file pone.0316276.s002.docx]

| ***MOHW-designated Joint IRB*** |
| --- |

**Study Protocol**

**(Human clinical trial)**

**ver 1.1**

Study title: Patient Registry study on Clinical Course Observation and Data Collection after Saegmaeksangagambang Administration in Patients with Hypertension

Patient Registry Study on the Clinical Course Observation and Data Collection of Saegmaeksangagambang for Hypertensive Patients

2023-HP- Saegmaeksangagambang

Investigator names and affiliations

| Name of investigator | Name of affiliation | Role |
| --- | --- | --- |
| Ho-Bin Moon | Kyung Hee Bichedam Korean Medicine Clinic | Principal investigator |
| Hyun-Sin Im | Kyung Hee Bichedam Korean Medicine Clinic | Sub-investigator |
| Chang-Seop Yang | Korean Institute of Oriental Medicine | Co-investigator |
| Kyung-Min Shin | Korean Institute of Oriental Medicine | Co-investigator |
| Byeong-Gap Kang | Korean Institute of Oriental Medicine | Co-investigator |
| Young-Eun Choi | Korean Institute of Oriental Medicine | Co-investigator |
| Jung-Tae Im | Wonkang University College of Korean Medicine | Co-investigator |
| Na-Hyun Cho | Wonkang University College of Korean Medicine | Co-investigator |

Table of Contents

[1. Study Background 6](#204099812)

[A. Isolated systolic hypertension (ISH) 6](#191151101)

[B. Effects of Saengmaeksan 7](#184509836)

[C. Definition of saengmaeksangagambang (modified SMS) 7](#184509838)

[D. Efficacy of individual ingredients of modified SMS [6] 8](#198281874)

[E. Effects of modified SMS in an animal study 11](#184509839)

[2. Purpose of study 12](#185102697)

[3. Names and positions of principal investigator, co-investigators, and sub-investigators 13](#185102695)

[4. Name and address of trial facility 13](#185102698)

[5. Sponsor 13](#185102699)

[6. Study period 14](#185102700)

[7. Study participants 15](#185102701)

[A. Recruiting facility 15](#185102712)

[B. Number of patients to be recruited 15](#185102713)

[C. Inclusion criteria 15](#185102714)

[D. Exclusion criteria 15](#185102715)

[E. Control group 15](#185197959)

[8. Anticipated sample size and grounds for sample size determination 15](#185102702)

[9. Participant recruitment 16](#185102703)

[10. Participant consent 16](#185102704)

[11. Method 17](#185102705)

[A. Outline of method 17](#185102736)

[B. Standard protocol for modified SMS 17](#185102759)

[C. Study intervention: administration of modified SMS per standard protocol 18](#185102738)

[D. Additional interventions, visits, and tests required for study participation per the purpose 19](#185102742)

[E. Control group, randomization, blinding 19](#185102743)

[F. Summary of study timeline (anticipated duration, place, staff in charge, study procedure) 20](#199018095)

[G. Management of human-derived materials 21](#185102746)

[12. Observation parameters 21](#185102706)

[A. Sociodemographic information 21](#204101099)

[B. History taking 21](#204101097)

[C. Measurement items 21](#185102855)

[D. Other 22](#185102931)

[13. Outcome measurement criteria and method 22](#185102707)

[A. Primary outcome 22](#185103118)

[B. Secondary outcome 22](#185103123)

[C. Outcome evaluation criteria 23](#185103129)

[14. Anticipated AEs, precautions, and measures taken to address them 23](#185102708)

[A. Additional AEs from participation in observational study 23](#185197865)

[B. Anticipated AEs from using modified SMS as part of routine treatment process 23](#185197870)

[C. Evaluation of AEs 23](#185197871)

[D. Analysis 23](#185197878)

[E. Reporting 23](#185197879)

[F. Treatment of AEs 24](#204101103)

[G. Evaluation of safety parameters 24](#185197869)

[15. Reporting of AEs 25](#204101104)

[A. Definition of AEs 25](#204101109)

[B. Serious adverse events (SAEs) 25](#204101110)

[C. Anticipated AEs and usage precautions 26](#204101111)

[D. Evaluation of AEs 26](#204101112)

[E. Reporting of AEs 27](#204101113)

[16. Data analysis and statistical methods 27](#185103139)

[17. Withdrawal of consent and discontinuation 28](#185103138)

[A. Discontinuation criteria 28](#185197890)

[B. Study termination criteria 28](#185197892)

[C. Withdrawal of consent 29](#185197889)

[18. Risks and benefits for study participants 29](#185102709)

[A. Potential risks 29](#185197898)

[B. Potential benefits 29](#185197899)

[C. Risk/benefits analysis 29](#185197900)

[19. Compensation for study participation 29](#185102710)

[20. Measures to protect participants’ safety and personal information 29](#185103140)

[21. Monitoring 30](#204101137)

[22. Reference 31](#185102711)

[23. Attachments 34](#204101452)

1. Study Background

It is well known that hypertension (HTN) substantially elevates cardio and cerebrovascular disease (CVD) incidence and mortality. A Korean study that followed male government employees and private school faculty and staff for six years showed that patients with HTN, defined as a blood pressure (BP) of 140/90 or higher, are at a 2.6-fold higher risk for CVD compared to those with a BP of below 130/85. The said study reported that HTN is the most potent risk factor for stroke. The risk for coronary artery disease (CAD) was 2.51 times higher among those with a BP of 130–140/85–90 mmHg and 5.08 times higher among those with a BP of 180/110 mmHg or higher compared to those with a BP of below 130/85 mmHg. Furthermore, the study revealed that the incidence of cerebral hemorrhage increases with increasing BP and that the risk for cerebral hemorrhage is significantly higher at a BP of greater than 130/85 mmHg. Moreover, another study has shown that HTN is an important factor in the onset of ischemic heart diseases.

The ultimate goal of HTN treatment is to prevent damages to the target organs. Study data have shown that every 5-mmHg drop in systolic blood pressure (SBP) is linked to a 34% drop in stroke risk and 21% drop in ischemic heart disease risk. Regarding herbal medicine, a number of different herbal medicine regimens, including hwangryeonhaedok-tang, have been found to be effective in lowering BP.

1. Isolated systolic hypertension (ISH)

ISH, a common condition observed in older adults, refers to a state characterized by high SBP and low diastolic BP (DBP); that is, it is defined as a DBP of below 80 mmHg and SBP of 130 mmHg or higher. This is the most common form of HTN among older adults aged 65 years and over, and young people can also develop ISH. Approximately 15% of individuals aged 60 years and older are estimated to have ISH. ISH can be caused by a variety of factors, including arteriosclerosis, hyperthyroidism, diabetes mellitus, heart valve disease, and obesity. Owing to the high mortality and morbidity rates among patients with chronic, uncontrolled ISH, ISH still remains an important public health problem.

1. Effects of Saengmaeksan (SMS)

Clinical trials have shown that herbal medicine prescriptions such as hwangryeonhaedok-tang, cheonghyeol-dan, samhwangsashim-tang, gyejibokryeong-hwan, jogudeung, bokbangdansam, cheonghyeon granules, daeshiho-tang, jinganshikpung-tang, banhabaekchulcheonma-tang, yukmijihwang-tang, ikgihwaeo-bang, pyeongganjamyang-bang, hyeolbuchukeo-tang, and bangpungtongseong-san, have been effective in treating HTN. SMS is a formula composed of ginseng, maekmundong, and Schisandra chinensis, and it has been traditionally used to boost qi and generate fluids and restrain yin to stop sweating. Its primary indications include ① fatigue and shortness of breath and dry throat and thirst caused by consumption of qi and damage of fluids from excessive sweating in the summer heat ② cough with little phlegm, shortness of breath with spontaneous sweating, and dry mouth and tongue due to damaged qi and yin from chronic cough and lung deficiency. In plain terms, its indications are fatigue and thirst due to excessive sweating and perspiration in summer, cough and phlegm, shortness of breath, spontaneous sweating, and dry mouth from long-term pulmonary diseases.

In vitro, SMS has been found to cardiovascular efficacy, including diabetic heart disease, diabetic autonomic impairment, myocardial ischemia, and arteriosclerosis. Furthermore, it is also prescribed for CAD [4] and angina [5] in clinical practice.

Clinical studies on the cardiovascular effects of SMS have predominantly studied improvements in stroke symptoms. In Korean medicine, gout, headache, vertigo, and increased liver yang are viewed as similar to the systemic symptoms of HTN, and the manifestation of pathological symptoms from HTN, a primary cause of stroke, is viewed to be closely related to the prodromal symptoms of stroke. Thus, the association between HTN treatment and SMS is worth studying.

One animal study have shown that SMS lowers BP, but no in vitro or clinical study findings exist for the BP-lowering effects of SMS.

1. Definition of saengmaeksangagambang (modified SMS)

In the present study, we modified the traditional SMS formula used for CVD (ginseng, Ophiopogon japonicus, and Schisandra chinensis) based on clinical experience, classic texts, and experiment findings. We removed ginseng and added Platycodon grandiflorus and Pueraria lobata as well as Dioscorea opposita and Coix lacrymajobi to stimulate digestive functions. The formula consisting of **Pueraria lobata (5.33g), Platycodon grandiflorus (5.33g), Ophiopogon japonicus (5.33g), Dioscorea opposita (2.67g), Coix lacrymajobi (2.67g), and Schisandra chinensis (2.67g) (total 24g; unit g/day)** is referred to as saengmaeksangagambang (modified SMS), and this prescription is empirically used at Kyung Hee Bi, Che, Dam Korean Medicine Clinic to treat CVD or autonomic nervous system symptoms. The ingredients are prepared as a decoction with alcohol as a solvent, in accordance with the concept of principal and ministerial inversion in Korean medical classic for use at the clinic. We will conduct an observational study using this prescription.

1. Effects of modified SMS in an animal study

There was an animal study showing that SMS was effective in BP regulation. In mice with normal BP, BP was lowered in the high-dose group, and local blood flow also increased dose-dependently in mice with normal BP given SMS. In a preclinical trial, SMS led to reduced NO production in rat aortic VSMC treated with TNF-α + IFN-γ, increased cell viability, and reduced iNOS protein and mRNA production.

A preclinical trial on the use of modified SMS (the investigational product of our trial) confirmed suppression of increased BP. A study on the effects of a three-week modified SMS regimen in mice with HTN induced with L-NAME showed that the BP was lowered to near-normal levels in the SMS group compared to the non-SMS group. In particular, ethanolic extract of modified SMS was more effective than water extract of modified SMS (Figures 2, 3).

In light of the result of the previously mentioned preclinical trial, where SMS reduced the production of vasoconstrictors in VSMC in mice, modified SMS is believed to be a promising therapeutic option for HTN.


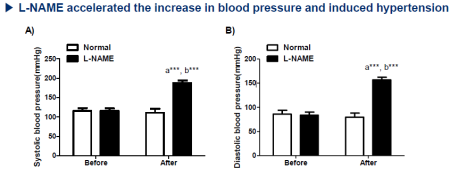


Figure 2

Graphs summarize systolic BP(A), and diastolic BP(B) (a,***p<0.001 Before L-NAME vs. After L-NAME ; b, *** p<0.001 After Normal vs. After L-NAME). BP was measured using the tail-cuff method. Results are expressed as the mean ±S.E. (n=6 mice per group). Before, L-NAME non-treatment; After, L-NAME treated.


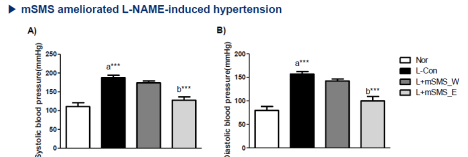


Figure 3

Graphs summarize systolic BP(A), and diastolic BP(B) in groups of Nor, L-Con, L+mSaengmaeksan_W, and L+mSaengmaeksan_E. Treatment with mSaengmaeksan_E ameliorated HTN in the L-Congroup (a, ***p＜0.001 Norvs. L-Con; b, ***p＜0.001 L-Convs. L+mSaengmaeksan_E). Results are expressed as the mean ±S.E. (n=6 mice per group). Nor, normal; L-Con, L_NAME control; L+mSaengmaeksan_W, mSaengmaeksan water extract treated L-NAME control groups; L+mSaengmaeksan_E, mSaengmaeksan ethanol extract treated L-NAME control groups.

1. Purpose of study

This study is an exploratory observational study that aims to explore the safety and BP-lowering effects of modified SMS and to collect HTN-related clinical parameters by comparing before and after administration of modified SMS, an herbal medicine prepared and prescribed at Kyung Hee Bi, Che, Dam Korean Medicine Clinic for the treatment of HTN.

1. Names and positions of principal investigator, co-investigators, and sub-investigators
2. Principal investigator (PI)

| Name | Affiliation | Job position | Contact information | Roles |
| --- | --- | --- | --- | --- |
| Ho-Bin Moon | Kyung Hee Bi,Che,Dam Korean Medicine Clinic | Director | 010-9326-1804 / realhobin@hanmail.net | PI, recruit and enroll participants, provide information about study and obtain consent, collect data, measure observation parameters, and perform tests |

1. Co-investigators

| Name | Affiliation | Job position | | Contact information | | Roles | |  |
| --- | --- | --- | --- | --- | --- | --- | --- | --- |
| Name | Affiliation | | Job position | | Contact information | | Roles | |
| Hyun-Sin Im | Kyung Hee Bichedam Korean Medicine Clinic | | Research assistant | | 010-3773-3302 / ohfeelgoods@naver.com | | Study administration and trial assistance | |
| Chang-Seop Yang | Korean Institute of Oriental Medicine | | Principal researcher | | 042-869-2705/yangunja@kiom.re.kr | | Study design | |
| Kyung-Min Shin | Korean Institute of Oriental Medicine | | Principal researcher | | 042-868-9604/kyungmin7221@kiom.re.kr | | Study design | |
| Byeong-Gap Kang | Korean Institute of Oriental Medicine | | Principal researcher | | 042-868-9557/bkkang@kiom.re.kr | | Develop data analysis and statistical analysis methods | |
| Young-Eun Choi | Korean Institute of Oriental Medicine | | Technical researcher | | 042-868-9410/wowo9129@kiom.re.kr | | Monitors | |
| Jung-Tae Im | Wonkang University College of Korean Medicine | | Assistant professor | | 010-9081-9908 / [julcho@naver.com](mailto:julcho@naver.com) | | Study design and data analysis consultatio | |
| Na-Hyun Cho | Wonkang University College of Korean Medicine | | Graduate student  (Master’s) | | 010-5036-9351 / [jnh528@naver.com](mailto:jnh528@naver.com) | | Assist in study design and data analysis | |

1. Roles by affiliation

* Kyung Hee Bi,Che,Dam Korean Medicine Clinic: recruit participants, collect informed consent, perform trial

* Korean Institute of Oriental Medicine: study design and analysis, monitoring, administration

* Wonkang University College of Korean Medicine: study design and data analysis consultation

1. Name and address of trial facility

Trial facility (single-center study): Kyung Hee Bi, Che, Dam Korean Medicine Clinic (28 Nonhyeon-ro 10-gil, Gangnam-gu, Seoul)

1. Sponsor

National Institute for Korean Medicine Development ([38540] 94 Hwarang-ro, Gyeongsan-si, Gyeongsangbuk-do (Gabje-dong))

1. Study period

Anticipated study period (1 year from approval by joint IRB. Scheduled to be completed on August 31, 2024)

1. Study participants
2. Recruiting facility

(Single-center) Kyung Hee Bi, Che, Dam Korean Medicine Clinic

1. Number of patients to be recruited

After screening candidates for eligibility, 30 participants who received mSMS will be recruited.

Study participants must meet all of the inclusion and not meet all of the exclusion criteria to be enrolled in the study.

1. Inclusion criteria
2. Adult men and women aged 19–74 years.
3. BP measured from the reference are at time of screening:

- If not taking antihypertensive drugs: 140 mmHg ≤ MSSBP(mean sittingSBP) < 180 mmHg
- If taking antihypertensive drugs: 130 mmHg ≤ MSSBP(mean sitting SBP) < 180 mmHg

1. Voluntarily provide a fully informed consent to provide medical records and personal information for use in the study.

- (*Reference arm is selected based on the process outlined in “12. Observation parameters-B.Measurement items”)
- (*Mean seated BP is defined in “12. Observation parameters-B.Measurement items)

1. Exclusion criteria
2. ≥ 20 mmHg difference in Systolic Sitting Blood Pressure (SSBP) and more than a 10 mmHg difference in Diastolic Sitting Blood Pressure (SDBP) across three consecutive measurements, taken at least 2 minutes apart in both arms
3. Mean seated SBP (MSSBP) ≥ 180 mmHg or mean seated DBP (MSDBP) ≥ 110 mmHg in the reference arm at time of screening
4. Liver disease or kidney disease requiring treatment
5. Secondary hypertension
6. History of severe CVD: e.g., myocardial infarction, angina, severe arrhythmia
7. Diagnosis of psychiatric disorder or treatment for psychiatric disorder, including depression, within 2 months of visit
8. Other reasons that contraindicate use of modified SMS as determined by the clinician

- (*Reference arm is selected based on the process outlined in “12. Observation parameters-B.Measurement items”)
- (*Mean seated BP is defined in “12. Observation parameters-B.Measurement items”)

1. Control group

A prospective single-group preliminary observational design; there is no control group, and blinding and randomization are not applicable.

1. Anticipated sample size and grounds for sample size determination

This is an exploratory preliminary prospective observational study. In general, preliminary studies require a minimum of 12 participants [9]. In the present study, we aim to obtain data from 30 participants.

F/u is performed every 4 weeks. In general, 67% of participants re-visit the facility on week 4.

To obtain data from 20 participants with a potential 33% dropout rate, **30 participants** are required.

Every month, about 10 patients take modified SMS, so recruiting the target sample size within the study period is feasible.

In preliminary studies, a minimum of 12 participants are generally required. **Given that this study is a preliminary study collecting exploratory data for subsequent studies, as opposed to a study investigating the therapeutic efficacy, recruitment of 30 participants in consideration of potential dropouts is deemed adequate.**

1. Participant recruitment

Participants will be recruited among patients who visit the Kyung Hee Bi, Che, Dam Korean Medicine Clinic and based on the clinician’s assessment and patient consent.

To ensure consistency of recruitment and fairness for potential participants, a recruitment announcement containing the overall information about the study, including its purpose, inclusion and exclusion criteria, and study schedule, will be posted on the bulletin board within the clinic for a specified period. Visitors to the healthcare facility interested in participating in the study can contact via the contact details listed in the announcement or by directly contacting the research team (PI and co-investigators).

Patients who express interest in participating will be informed about the study’s purpose, methods, and that there is no additional financial burden for treatment and testing. Those who provide voluntary consent will be enrolled.

The participant recruitment process is as follows: patients with HTN visiting the study facility who qualify per the eligibility criteria and provide consent will be enrolled in the study as part of their treatment process, and their anthropometry and changes in BP and quality of life will be monitored using tests and questionnaire; thus, the participants will be consecutively recruited.

The names and contact details of participating patients will be accessible only to the research team. The PI and sub-investigator will contact each participant individually to arrange their participation schedule.

1. Participant consent

- **Before beginning observational study** The purpose and details of this observational study will be explained in detail, along with the tests and treatments performed as part of the study.

- The informed consent form is collected only when the study participants are given adequate time to understand the purpose, method, risks, and benefits of study participation, and oluntarily agree to participate. - At the initial visit to the healthcare facility, the research team will obtain the informed consent from the participants and enroll them.

- The PI, Ho-Bin Moon (Korean medicine doctor), will obtain the informed consent forms.

- Participants do not consent to participate, do not qualify per the eligibility criteria, do not take the modified SMS even once during the observation period even after signing informed consent form and qualifying per eligibility criteria, or refuse to participate during the study period may be **discontinued from the study.**

-The informed consent form will be obtained in a quiet, private space where the investigators can communicate with the study participants without interruptions; it will be a private space within the Kyung Hee Bi, Che, Dam Korean Medicine Clinic. The names and contact information of the participants will only be accessible to the research team.

- The participants are informed that they will not receive any form of disadvantage or impact on their treatment for not participating in the study.

- The study is conducted under a national research and development project commissioned by the National Institute for Korean Medicine Development (NIKOM) (evidence-generating research as part of Korean medicine innovative technology development project). The research data collected according to the study announcement will be recorded and stored through E-CRF (Mytrial) established by NIKOM and provided to them. Provision of the study data to a third party and use of the data for secondary studies will be permitted only after obtaining a separate consent from from the participants for the purpose.

1. Method
2. Outline of method
3. Study period

Anticipated study period (1 year from approval date. Scheduled to be completed by August 31, 2024)

1. Study site

Kyung Hee Bi, Che, Dam Korean Medicine Clinic; a prospective observational study on patients

1. Study design

Single-group, prospective, exploratory, preliminary observational study

This study is an observational study for preliminary exploration of the therapeutic effects of modified SMS on HTN and for exploration of feasibility for future randomized controlled trial.

1. Study schedule (standard management protocol): 4 clinical assessments

Baseline (wk 0, screening and enrollment, visit 1)

Mid-treatment (4 weeks after beginning dosing of modified SMS) (visit2)

End of treatment (8 weeks after beginning dosing of modified SMS) (visit3)

4-week f/u after treatment (12 weeks after beginning dosing of modified SMS) (visit4)

1. Standard protocol for modified SMS

The participants will undergo the following process per the standard protocol:

When using modified SMS, those receiving any other KM treatments, including acupuncture, pharmacopuncture, and moxibustion, will be excluded

1. Standard herbal medicine prescription protocol

- Compliance with pre-visit instructions:
  After making an appointment for the visit, patients are instructed to fast for 8 hours before the visit, refrain from smoking, drinking, and consuming caffeine the day before the visit, and to visit the clinic between 8–12 AM.
- History taking
- 3D BP waveform test (3-d pulse imaging study), blood test
- Same tests performed at F/u on weeks 4, 8, and 12 after herbal medicine prescription

1. Standard protocol visits

- (Visit) Patients who take herbal medicine prescribed by Kyung Hee Bi, Che, Dam Korean Medicine Clinic are instructed to make f/u visits every 4 weeks for monitoring of AEs and clinical progress. In case of AEs, additional visits may be necessary.

1. Standard protocol tests

- The Standard management protocol at Kyung Hee Bi, Che, Dam Korean Medicine Clinic involves performing 3D BP waveform test and blood tests on patients taking herbal medicines every four weeks. In the present study, only patients taking modified SMS were enrolled. However, additional tests may be performed in cases of AEs.

1. Study intervention: administration of modified SMS per standard protocol

In the present study, we modified the traditional SMS formula used for CVD (ginseng, Ophiopogon japonicus, and Schisandra chinensis) based on clinical experience, classic texts, and experiment findings. We removed ginseng and added Platycodon grandiflorus and Pueraria lobata as well as Dioscorea opposita and Coix lacrymajobi to stimulate digestive functions. The formula consisting of **Pueraria lobata (5.33g), Platycodon grandiflorus (5.33g), Ophiopogon japonicus (5.33g), Dioscorea opposita (2.67g), Coix lacrymajobi (2.67g), and Schisandra chinensis (2.67g) (total 24g; unit g/day)** is referred to as saengmaeksangagambang (modified SMS). This is one of the unique empirical prescriptions used at Kyung Hee Bi, Che, Dam Korean Medicine Clinic. Although in actual practice at Kyung Hee Bi, Che, Dam Korean Medicine Clinic, **certain ingredients may be removed, doses may be adjusted, or other interventions such as acupuncture, may be added depending on the symptoms and disease being treated, such cases were excluded from the analysis in this study.**

- Patients take a 60-mL 70% alcohol-soaked decoction of the modified SMS formula prepared by Kyung Hee Bi, Che, Dam Korean Medicine Clinic twice daily before meals or between meals
- Dispensing of medicine on Visit 1, Visit 2: [number of days until next scheduled visit date (standard 28 days, maximum 35 days) + 3 days]* 2 packs. A standard of 62 packs, up to 76 packs, are packaged in a box or pack, labeled with the patient’s information.
- Participants will return unused medicine at the subsequent visit.
- Returned unused medications will be stored separately for each patient in a box/pack.
- At completion of trial, returned medicines will be disposed of following the facility’s medication disposal procedures.
- Dose per pack: modified SMS: each pack contains 60 mL containing Pueraria lobata (5.33g), Platycodon grandiflorus (5.33g), Ophiopogon japonicus (5.33g), Dioscorea opposita (2.67g), Coix lacrymajobi (2.67g), and Schisandra chinensis (2.67g) (total 24g; unit g/day).
- Dosage: oral; 2 packs a day
- Treatment period: 8 weeks (56 days) (f/u every 4 weeks)
- Permitted concomitant treatments: Given the observational nature of this study, we will not limit any concomitant treatments and medications while undergoing the SMS regimen, and concomitant treatments will be documented.
  * Use/non-use of antihypertensive drugs will be documented.
  * If antihypertensive drugs are being used, patients are informed to notify the investigator upon changes in the dose and dosage of the drug.
  * Patients are informed to maintain their normal lifestyle, including exercise and diet.
- The dose and dosage of modified SMS used in this trial are identical to those used in actual practice at Kyung Hee Bi, Che, Dam Korean Medicine Clinic.

|  | | |
| --- | --- | --- |
| 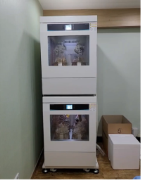 | 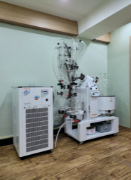 | 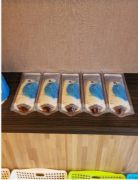 |
| Cold extraction | Vacuum concentration | Concentrate |

Figure 5. Preparation facilities at Kyung Hee Bi, Che, Dam Korean Medicine Clinic and appearance of concentrates

1. Additional interventions, visits, and tests required for study participation per the purpose

The Standard management protocol at Kyung Hee Bi, Che, Dam Korean Medicine Clinic involves performing 3D BP waveform test and blood tests on patients taking herbal medicines every four weeks. In the present study, only patients taking modified SMS were enrolled. **Thus, there are no additional interventions required for participation in this study**

**In case of an adverse event, f/u is needed until the adverse event is resolved. In such cases, additional visits may be necessary.**

In case of an adverse event, additional tests, such as kidney function test, liver function test, and complete blood count, may be performed.

1. Control group, randomization, blinding

This is a single-group study without a control group, so randomization and blinding are not applicable.

1. Summary of study timeline (anticipated duration, place, staff in charge, study procedure)

A study questionnaire will be used to obtain data, and a total of five test parameters will be monitored. The questionnaire and tests will be performed at every visit, and it will take about 30 minutes. The questionnaire and tests will be performed at Kyung Hee Bi, Che, Dam Korean Medicine Clinic, and the PI will be in charge of distributing and collecting the questionnaire and performing tests.

| Item | Screening and enrollment | Treatment phase | | F/U |
| --- | --- | --- | --- | --- |
|  |  | 4wks | 8wks | 12wks |
| Visit | Visit 1 | Visit 2 | Visit 3 | Visit 4 |
| Visit window | 0주 | Week 4 (±7 days) | Week 8  (±7 days) | Week 12  (±7 days) |
| Informed consent | ○ |  |  |  |
| Sociodemographic information | ○ |  |  |  |
| VS and physical examination | ○ | ● | ● |  |
| Compliance with pre-visit instructions | ○ | ● | ● |  |
| Medical history·treatment history·medication history | ○ |  |  |  |
| Inclusion and exclusion criteria | ○ |  |  |  |
| Blood test | ● | ● | ● |  |
| BP and pulse measurement | ○ | ● | ● |  |
| 3D BP waveform test | ● | ● | ● |  |
| European Quality of Life Five Dimension Five Level Scale (EQ-5D-5L) | ● | ● | ● |  |
| Modified SMS prescription and dispensing | ● | ● |  |  |
| Monitoring of herbal medicine adherence and dosage |  | ● | ● |  |
| Monitoring of AEs |  | ● | ● | ● |
| Changes in Medical history·treatment history·medication history ** |  | ● | ● | ● |
| Conclusion of case* |  |  |  | ● |

Table 2. Summary of study data collected by timeline

* Conclusion of case: collected after visits are completed or participant is discontinued

** Changes in Medical history·treatment history·medication history: Collect information upon changes in Medical history·treatment history·medication history since the visit

● Tests performed only on those who qualify per the inclusion/exclusion criteria

eCRF will be identical to the paper-based CRF, and the Mytrial program at NIKOM will be used to establish the eCRF.

Modified intervention records: if the treatment interventions are changed mid-way, the changes will be recorded in the modified intervention record section of the CRF.

Concomitant drugs and treatment: Concomitant drugs and treatment will be recorded in the respective section in the CRF.

1. Management of human-derived materials

In this study, the results of blood tests performed as part of the standard management protocol used at the study clinic will be analyzed. Thus, no additional blood tests will be performed due to the study, and results from the blood tests performed routinely on the patients per their treatment protocol will be collected and analyzed for the purpose of this study.

Collection of human-derived materials (blood): 35 ㎕ of blood will be collected through a finger puncture and immediately placed in the kit for analysis using a LABGEO PT10 Analyzer.

Analysis of human-derived materials (blood): blood sample will be analyzed using a LABGEO PT10 Analyzer

Disposal of human-derived materials (blood): After analysis, the blood will be placed in a biohazard waste container and will be discarded with other biohazard wastes twice a month by a biohazard waste removal company (Munhwa Green, 02-3461-1933).

1. Observation parameters
2. Sociodemographic information
3. History taking
4. History
5. Surgical history
6. Medication history

* Information about antihypertensive medication use will be collected.
* If the patient is taking antihypertensive drugs, they are instructed to notify the investigator regarding the dosage of the drug and any changes to the dosage.
* Patients are informed to maintain their normal lifestyle, including exercise and diet.

1. Measurement items
2. Height and VS (excluding BP and pulse): 5 minutes of measurement followed by 15-min rest
3. BP and pulse
   - - 1. Measurement device: electronic blood pressure cuff Omron Healthcare (Model: HEM-9000AI)
       2. Measurement items

- MSSBP (Unit: mmHg)
- MSDBP (Unit: mmHg)
- Pulse (Unit: beats/min)
  - - 1. Measurement method
- **Definition of MSBP:** the average of **three or more measurements** taken with at least 2 minutes apart is used. BP is measured in a straight seated position (seated BP).
- **Arm selection**: BP is measured from both arms if possible, and the arm with the higher BP is selected. Subsequent BP measurements must be taken from the same arm.
- Posture should remain unchanged while measuring BP. A supine or seated position must be maintained for at least five minutes, and a standing posture must be maintained for at least one minute before taking the measurements.

1. Blood test
   - - 1. Measurement device: LABGEO PT10 Analyzer
       2. Measurement items: Aspatate transaminase(AST), Alanine transaminase(ALT), Gamma glutamyl peptidase(GGT), Total Bilirubin, BUN, Creatinine, Total cholesterol, Glucose
       3. Measurement method

- Refrain from caffeine, alcohol, and tobacco consumption the day before the test
- 8-hour fasting is required before the test; water allowed
- Test takes 10 minutes

1. [**3D Blood pressure and pulse wave test]**: **3d pulse wave imaging device (3D MAC, ㈜ Daeyomedi Co., Ltd., South Korea)**: 10 minutes ( See [Attachment 1])
   - - 1. Pulse strength value and stage (Unit:gf)
       2. Pulse depth value and stage (Unit:gf/cm^2)
       3. Pulse velocity value and stage (Unit:beats)
       4. Pulse shape value and stage (Unit:step)
       5. Radial artery augmentation index(R-AI) (Unit:%)
       6. SBP (Unit:mmHg)
       7. DBP (Unit:mmHg)
       8. Pulse pressure (PP) (Unit:mmHg)
       9. Pulse rate (PR) (Unit:beats/min)
       10. Cardiac output (1 beat) SV (Unit:mL/beat)
       11. Cardiac output (1 minute) CO (Unit:L/min)
       12. Systemic vascular resistance index(SVRI) (Unit:dyn*s/cm^5/m^2)
2. European Quality of Life Five Dimension Five Level Scale (EQ-5D-5L) questionnaire: 5 minutes ( see [Attachment2] )
3. Other

Adverse events and concomitant treatments are monitored per patient’s report and questions at every visit

1. Outcome measurement criteria and method
2. Primary outcome

This study is an exploratory observational study aiming to explore the safety and BP-lowering effetcs of modified SMS prepared and prescribed by Kyung Hee Bi, Che, Dam Korean Medicine Clinic for patients with HTN by comparing SBP before and after treatment.

1. [Changes in MSSBP] use Omron Healthcare HEM-9000AI
   Amount of change in MSSBP at end of treatment (visit 3) compared to the baseline (visit 1)
   - - 1. Null hypothesis (H0): There is no difference in MSSBP between the baseline (Visit 1) and end of treatment (visit 3).
       2. Alternative hypothesis (HA): There is a difference in MSSBP between the baseline (Visit 1) and end of treatment (visit 3).
2. Secondary outcome
3. Amount of change of MSSBP at visit 2 compared to the baseline (visit 1)
4. Amount of change of MSSBP at visits 2 and 3 compared to the baseline (visit 1)
5. Percentage of participants with normalized BP: percentage of patients who achieved normal BP compared to MSSBP/MSDBP measured at each time point (percentage of patients who had a BP of less than 140/90 mmHg at end of treatment)
6. BP response rate at end of treatment (visit 3) compared to the baseline (visit 1) (percentage of patients who had MSSBP drop by 20 mmHg or more or MSDBP drop by 10 mmHg or more)
7. Amount of change in individual parameters on 3D pulse wave imaging at visits 2 and 3 compared to the baseline (visit 1)
8. Amount of change in EQ-5D-5L score at visits 2 and 3 compared to the baseline (visit 1)
9. Outcome evaluation criteria

This study is a prospective study conducted to present data for subsequent studies on modified SMS; the purpose is not to determine the clinical efficacy of the drug, but rather, to explore and collect preliminary data.

However, for the statistical analysis in this study, we will present the degree of improvement in the continuous variables using descriptive statistics.

Efficacy is determined based on the improvement of MSSBP, 3D pulse wave imaging parameters, and Eq-5D-5L score at visits 2 and 3 compared to the baseline, and the percentage of patients who have achieved such improvements is presented. However, because the study is an exploratory study without a control group, there are limitations in determining efficacy.

As this study observes effects during a routine treatment protocol, we will collect the following data that may potentially influence BP and control for them in the analysis.

* Collection of information about the use of antihypertensive drugs.
* If the patient is using antihypertensive medication, they are informed to notify the investigator upon changes in the dose and dosage of the drug.
* Patients are informed to maintain their normal lifestyle, including exercise and diet

ANCOVA is performed with sociodemographic variables that may affect HTN, namely sex, age, BMI, duration of HTN, comorbidity, BP at initial visit, use of antihypertensive medication, and types of drugs, controlled for as covariates.

1. Anticipated AEs, precautions, and measures to address them
2. Additional AEs anticipated as a result of participation in observational study

In this observational study, the same process used in the standard treatment protocol is used, which involves taking modified SMS, performing tests and questionnaires, and documenting them in the CRF. Thus, there are no additional tests or interventions performed other than those included in the standard treatment process; for this reason, there are no other anticipated AEs in addition to the AEs that may occur during the standard treatment process.

1. Adverse events anticipated from use of modified SMS during routine treatment process

There is a low risk for AEs anticipated due to the use of modified SMS (risk for adverse effects and tolerance to modified SMS), but there is a risk for the following AEs that may occur with the routine treatment protocol:

- Gastrointestinal symptoms (Indigestion, heartburn)
- Autonomic nervous system symptoms (palpitation)

Thus, other than the AEs associated with the use of modified SMS for therapeutic purposes, there is little to no anticipated risks or adverse effects from study participation.

1. Usage precautions

- The participants are educated about the following precautions when taking the herbal medicine:
- Keep the herbal medicine in a cool place or in the refrigerator.
- Once opened, take the herbal medicine as soon as possible, and any opened herbal medicine must be kept in the refrigerator within two days.

1. Education on AEs

The PI and co-investigators of the observational study will inform the participants or their caregivers about the potential AEs following the use of modified SMS during the study period and explain that there is little risk for additional AEs from study participation because the study does not involve additional intervention or tests other than those included in the routine treatment protocol. The participants should also be informed to contact the research team immediately upon developing an AE.

1. Collection and reporting of AEs

At every visit to the Kyung Hee Bi, Che, Dam Korean Medicine Clinic, VS measurements, examination, and questionnaire for the observation parameters, or monitoring of AEs will be performed. If the investigator finds that the participant has clinically significant symptoms or changes and if the study participant voluntarily reports such an event, the investigator will document the incidence, evaluation, and measures for AEs in the CRF using standardized medical terminology.

In case of a serious adverse event (SAE) during study participation, the SAE will be promptly reported via the e-IRB system in accordance with Article 4–25 of the Standard Operating Procedures of the joint IRB. SAE is defined per the Standard Operating Procedures of the joint IRB.

1. Treatment of AEs

If a participant incurs a direct injury from the use of modified SMS as part of the routine treatment process, appropriate medical treatment will be given based on the routine treatment process.

This study is an observational study collecting clinical data and observing the clinical course of patients taking modified SMS, and there are no interventions other than the herbal medicine regimen or any additional tests required for study participation. If an AE occurs nonetheless, the herbal medicine regimen will be discontinued, and the patient will be observed and treated as necessary until the event is resolved per the general treatment protocol at Kyung Hee Bi, Che, Dam Korean Medicine Clinic.

Measures taken for abnormal blood test results: liver function test including aspartate transaminase (AST), Alanine transaminase (ALT), Gamma glutamyl peptidase (GGT), and total bilirubin, as well as BUN, creatinine, total cholesterol, and glucose are measured on visits 1, 2, and 3. Per the general treatment protocol at Kyung Hee Bi, Che, Dam Korean Medicine Clinic, additional use of herbal medicine regimen is suspended if the levels rise more than twofold, along with the standard medical measures (including tests) for f/u.

Given the minimal anticipated side effects, the study is not expected to have substantial safety issues. As a rule, safety parameters are monitored, and any incidence of AEs will be notified to the PI and co-investigators. Upon onset of an AE, appropriate medical measures will be taken until recovery.

1. Evaluation of safety parameters

Safety evaluation will be performed for all AEs that occur during the study period. The rate of AE, rate of AE that led to discontinuation, and rate of SAE are reported. For the rate of AEs, all AEs and AEs associated with Korean medical care and intervention will be presented.

VS or blood test results will be reviewed comprehensively to determine normal/abnormal results, and items deemed clinically significant by the clinician can be analyzed statistically as necessary.

1. Data analysis and statistical methods

Efficacy evaluation will be performed using the FAS. Safety data will be analyzed with the safety set. All analyses will be conducted using the SAS Version 9.4 (SAS Inc, Cary, NC) software, and all statistical tests not defined separately will be performed as a two-tailed test and 5% significance level. Any missing values in the FAS will be imputed using the Last Observation Carried Forward (LOCF) method for the efficacy evaluation. All other data will be analyzed as is.

Full Analysis Set(FAS): Per the Intention-to-Treat (ITT) principle, FAS includes participants who have received the herbal medicine preparation at least once and have had at least one measurement of the primary outcome measure

Safety Set (SS): Includes participants who have received the herbal medicine preparation at least once and have had at least one safety-related f/u

For demographic and baseline data, categorical data will be presented as n (%), and continuous data will be presented as mean±SD.

For efficacy parameters, continuous variables will be analyzed with paired t-test or Wilcoxon signed rank test, and categorical variables will be analyzed using the McNemar test or McNemar Exact test depending on their normality.

For safety parameters, adverse drug reactions (ADRs), SAEs, and serious ADRs will be presented as n (%), and differences across time points will be analyzed using the McNemar test or McNemar Exact test.

ANCOVA model will be used for analysis with sociodemographic factors that may affect HTN, such as sex, age, and use of antihypertensive drugs, included as covariates.

1. Withdrawal of consent and discontinuation
2. Discontinuation criteria

Participants may be discontinued from the trial in the following instances:

- Participants or their legal representatives requests discontinuation or participant withdraws consent due to unsatisfactory treatment effects during the study period
- Violation of inclusion criteria or meeting exclusion criteria
- Failure to take the modified SMS even once during the observational study period, despite giving consent and meeting eligibility criteria
- Occurrence of SAE related to the herbal medicine or AE making continuation in the study difficult
- Change of prescription during study
- Participant’s violation of study plan
- Lost to f/u during study period
- Other reasons due to which continuation of study is deemed inappropriate by the investigator

1. Study termination criteria

Upon occurrence of SAE suspected to be caused by the investigational drug, the PI notifies the joint IRB within 24 hours and terminate the study.

The clinical trial may be terminated prematurely in the following cases:

- Harmfulness of investigational product is discovered
- Moderate or severe AE deemed to have a relationship with the clinical trial occurred in more than 25% of the entire study population
- Study poses threat to the safety and well-being of study participants

1. Withdrawal of consent

Even after voluntarily consenting to participate, participants may withdraw their consent at any time without any disadvantages. If a participant wishes to withdraw their consent or meets the criteria for study discontinuation, their personal information and relevant data will be excluded from the analysis and will be discarded immediately.

1. Risks and benefits for study participants
2. Potential risks

The study lacks a control group, and only routine intervention and tests are involved; thus, there is no added risk from study participation.

During routine treatment, modified SMS has little risk for side effects or tolerance, but gastrointestinal symptoms (indigestion, heartburn) and autonomic nervous system symptoms (palpitations) may occur.

Since no additional interventions or tests are involved, there will be no cases of injuries caused by participation in the study. However, issues arising from routine medical care can be compensated according to the clinic’s malpractice liability insurance policy.

1. Potential benefits

Since the treatment protocol for modified SMS at Kyung Hee Bi, Che, Dam Korean Medicine Clinic is followed, there is no direct benefit to participants from participating in the study.

However, by participating in the study, participants contribute to gathering information on the effectiveness of modified SMS in improving HTN and observing its progress, which can aid in the future development of Korean Medicine-based treatments for HTN.

1. Risk/benefits analysis

Considering the potential risks and potential benefits, there is a low risk of side effects and no additional benefits to treatment. However, there are also no additional risks posed by study participation. Thus, the study is deemed to pose minimal risk to the participants.

1. Compensation for study participation

The compensation for the study participants (e.g., transportation) is 50,000 KRW for each visit (1, 2, 3) for a total of 150,000 KRW. Visit 4 is a telephone f/u, so there is no compensation.

Participants’ bank account numbers will be additionally collected for payment of the compensation.

1. Measures to protect participants’ safety and personal information
2. Information collected for routine medical care and patient management

Information collected for routine medical care and patient management includes personal information (name, resident registration number, email, phone number), demographic information (e.g., age, sex), medical history (e.g., past medical history, medication history, surgical history, family history), and various clinical outcomes (BP, height, weight, 3D blood pressure waveform test, EQ-5D-5L score). In addition, other details described in the observation items typically collected in Korean medicine practice [20] are collected. These do not deviate from the scope of information collected in routine medical care.

This study is a part of the national project commissioned by the NIKOM, and accordingly, study data collected according to the recruitment announcement will be documented and saved via eCRF (mytrial) established by the NIKOM and provided to the NIKOM. The data will only be provided to third parties or used for secondary research with the explicit informed consent of the participants.

1. Information additionally collected for study purposes

In addition to the information collected during routine medical care for study purposes, the participant’s bank account information will be additionally collected for payment of compensation.

1. Confidentiality

Personally identifiable data will be kept confidential, and participants will remain anonymous even when the results of the observational study are published. The details are as follows.

The investigators must be aware that upon the signing of the contract for this observational study, the Sponsor or auditor may review or copy the participant's charts and CRF for verification purposes.

1. Study data storage method and place

Relevant data will be stored in a locked, protected place (locked cabinet in the investigator’s office or password-protected computer) to ensure access to only permitted individuals.

In all study-related documents, participant identification codes (participant initials) will be used instead of their names.

1. Retention period

Personal information will be discarded three years after study completion

In accordance with Article 15 of the Enforcement Regulations of the Act on Bioethics and Safety, study data (IRB decision, written informed consent, personal information collection/use/provision status, study completion report) will be retained for three years after the study completion

1. Disposal method

In accordance with Article 15 of the Enforcement Regulations of the Act on Bioethics and Safety, personal information will be shredded immediately after study completion.

Study data will be shredded by a shredding service three years after completion of the study to prevent any leakage of information.

Information provided to the NIKOM via Mytrial will be retained for 10 years after study completion.

1. Monitoring

- Monitoring is performed to protect the rights and welfare of the participants, to ensure the reported clinical research data is accurate, complete, and verifiable against source documents, and to confirm that the trial is performed in accordance with the approved protocol, clinical trial management standards, and enforcement regulations. The monitoring is performed by a NIKOM-appointed monitor in the forms of regular site inspections or phone checks. During visits, monitors review participant records, inventory management records, and data storage (study files). The scope of monitoring activities includes ensuring compliance with the clinical trial protocol, checking for accurate and appropriate data collection, reviewing participant consent and re-consent forms, and reviewing collection and reporting of (serious) AEs and management of investigational products.
- Monitoring visits in this clinical study are categorized into initial, periodic monitoring, and end-of-study monitoring. The first monitoring visit occurs within 3 weeks after the enrollment of the first study participant. Periodic monitoring visits are scheduled based on the pace of participant recruitment, and end-of-study monitoring visit will occur after the end-of-study visit of the last participant. Additional monitoring visits may be conducted during the study period for purposes such as managing safety information, addressing low participant recruitment, or non-compliance with the study protocol or relevant regulations and guidelines.
- Monitors closely observe the progress of the clinical study and consult with investigators if any issues arise. The timing of their visits is coordinated between the investigators and the monitors. The investigators are also required to provide access to essential documents, such as participant source documents and basic study documents, as defined in the clinical study management standards and regulations, such that the monitors can verify the data entered in the CRF.
